# Case Report: *BMPR2*-Targeted MinION Sequencing as a Tool for Genetic Analysis in Patients With Pulmonary Arterial Hypertension

**DOI:** 10.3389/fcvm.2021.711694

**Published:** 2021-09-13

**Authors:** Tomoya Takashima, Sophie Brisset, Asuka Furukawa, Hirohisa Taniguchi, Rika Takeyasu, Akio Kawamura, Yuichi Tamura

**Affiliations:** ^1^Pulmonary Hypertension Center, International University of Health and Welfare, Mita Hospital, Tokyo, Japan; ^2^Faculty of Medicine, Université Paris-Saclay, Le Kremlin-Bicêtre, France; ^3^Service d'Histologie, Embryologie et Cytogénétique, Assistance Publique Hôpitaux de Paris (AP-HP), Hôpital Antoine Béclère, Clamart, France; ^4^Department of Cardiology, International University of Health and Welfare School of Medicine, Narita, Japan

**Keywords:** nanopore sequencing, MinION, BMPR2, PAH, next generation sequencing

## Abstract

**Background:** Mutations in the bone morphogenetic protein receptor type 2 gene (*BMPR2*) represent a major genetic cause of pulmonary arterial hypertension (PAH). Identification of *BMPR2* mutations is crucial for the genetic diagnosis of PAH. MinION nanopore sequencer is a portable third-generation technology that enables long-read sequencing at a low-cost. This nanopore technology-based device has not been used previously for PAH diagnosis. This study aimed to determine the feasibility of using MinION nanopore sequencing for the genetic analysis of PAH patients, focused on *BMPR2*.

**Methods:** We developed a protocol for the custom bioinformatics pipeline analysis of long reads generated by long-PCR. To evaluate the potential of using MinION sequencing in PAH, we analyzed five samples, including those of two idiopathic PAH patients and a family of three members with one affected patient. Sanger sequencing analysis was performed to validate the variants.

**Results:** The median read length was around 3.4 kb and a good mean quality score of approximately 19 was obtained. The total number of reads generated was uniform among the cases and ranged from 2,268,263 to 3,126,719. The coverage was consistent across flow cells in which the average number of reads per base ranged from 80,375 to 135,603. We identified two polymorphic variants and three mutations in four out of five patients. Certain indel variant calling-related errors were observed, mostly outside coding sequences.

**Conclusion:** We have shown the ability of this portable nanopore sequencer to detect *BMPR2* mutations in patients with PAH. The MinION nanopore sequencer is a promising tool for screening *BMPR2* mutations, especially in small laboratories and research groups.

## Introduction

Pulmonary arterial hypertension (PAH) is a rare, severe, and progressive disorder associated with vascular remodeling and the narrowing of small pulmonary arteries. The increase in pulmonary vascular resistance can result in right heart failure and eventual death (1–3). Pulmonary arterial hypertension may be inherited from an affected relative (heritable PAH) or induced by a drug, toxin, or associated with another disease (congenital heart disease, connective tissue disease, HIV). However, in most cases of PAH, there is no identifiable cause or family history of disorder (idiopathic PAH) (4).

Developments in genetics and technological advances have contributed to our knowledge of this lethal disease. Most heritable PAH cases are caused by a mutation in the bone morphogenetic protein receptor type 2 gene (*BMPR2*), a receptor for the transforming growth factor-beta (TGF-β) superfamily (5, 6). *BMPR2* mutations are detected in approximately 80% of individuals with family history and in 11–40% of patients with sporadic PAH (7). The rate of occurrence of *BMPR2* mutations in Japanese patients is the same as that reported in other countries. Mutations have been identified less frequently in other genes, including *ACVRL1* (also known as *ALK1*), *ENG, TBX4, KCNK3, CAV1*, and *SMAD9*, among others (8, 9).

The identification of *BMPR2* mutations is a crucial step in the genetic diagnosis of PAH. It has implications for personalized therapy and genetic counseling (7–11). The usual approach is to perform Sanger sequencing, but the process is time consuming and labor-intensive. The complexity, efficiency, and rapidity of sequencing methods continues to increase. Next-generation sequencing (NGS) technology transformed the manner in which sequencing was performed (12). Next-generation sequencing exhibited a higher sensitivity in the detection of novel and rare variants (9). The past few years have seen the emergence of a nanopore-based third-generation sequencing technology, known as nanopore DNA sequencing (13). The low-cost and rapid MinION-based nanopore sequencing method has emerged as a competitive and portable technology that has provided accurate long sequence reads (14). Here, we developed a rapid method for genetic analysis that employed MinION technology through a custom analysis pipeline. Because of the high incidence of *BMPR2* mutations, we particularly focused on this gene. To the best of our knowledge, this article is the first to report about the use of MinION for PAH genetic analysis.

## Materials and Methods

### Patients and Genomic DNA

Five samples, including those of two patients with idiopathic PAH (Cases #1 and #2) and a family of three (Cases #3, #4, and #5) were investigated in this study. In the family, only the father (#3) was diagnosed with PAH; the siblings, a daughter (#4) and a son (#5), were unaffected. This study was approved by a local institutional ethics committee at the International University of Health and Welfare (approval number 5-16-25); informed consent was obtained from each patient. Whole blood samples were collected in EDTA-containing tubes and sent to a commercial laboratory (SRL, Tokyo, Japan) for DNA extraction, according to standard procedures. After genomic DNA extraction, DNA samples were stored in the laboratory at 4°C. The integrity of the DNA was checked using a NanoDrop One Spectrophotometer and the DNA was diluted to a concentration of 20 ng/μl.

### Long-PCR Target Enrichment

**Figure 1:**
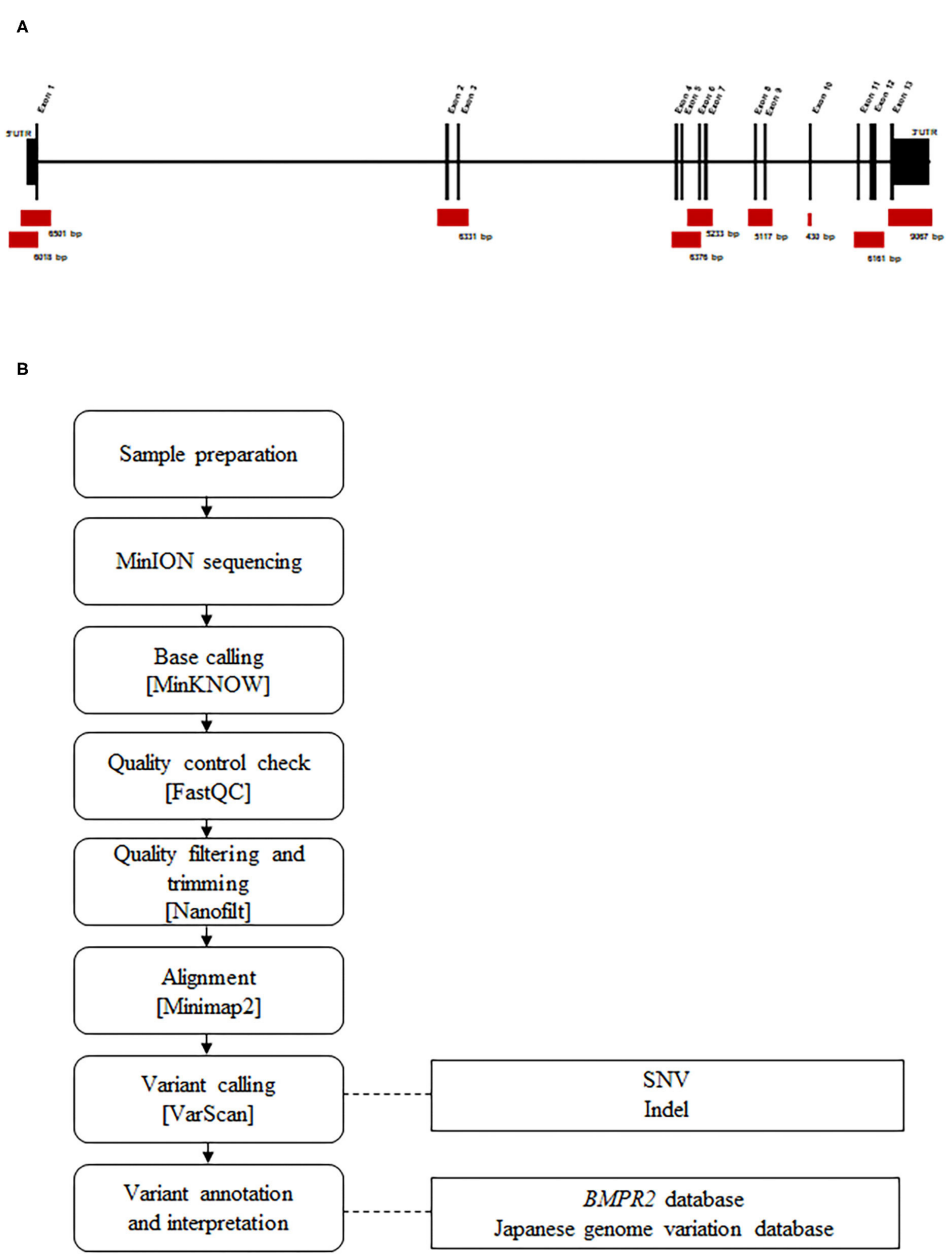
Design and workflow of the *BMPR2* targeted MinION sequencing process. **(A)** Representation of the *BMPR2* genomic structure and location of the primer sets used for long-PCR amplification. **(B)** Sequencing workflow and the custom bioinformatics pipeline.

Before sequencing, DNA was amplified by targeted long-PCR. Primers were designed using Primer-BLAST to amplify the *BMPR2* exonic sequences (including UTR regions) and surrounding intronic sequences (adjacent, upstream, and/or downstream exonic sequences). The primer locations and corresponding amplicon sizes are presented in Figure 1A. Details regarding the primer sequences and conditions are described in Supplementary Table 1.

We used a long-PCR based strategy with *BMPR2*-specific primers tailed with universal sequences, as recommended by Oxford Nanopore. PCR was performed using the KAPA HiFi HotStart ReadyMix PCR Kit (KAPA BIOSYSTEMS) on a T100 Thermal Cycler (BIO-RAD).

The amplification quality was checked via agarose gel electrophoresis and PCR products were purified using the QIAquick PCR Purification Kit (QIAGEN), according to the manufacturer's protocol.

### Library Preparation and Nanopore Sequencing

Library preparation was performed using the protocol 1D amplicon/cDNA, by ligation (SQK-LSK109) (Oxford Nanopore). Sequencing was performed using the MinION device, along with a FLO-MIN106 (Pore type R9.4) Flowcell. The MinION device was controlled by the MinKNOW software during the run time of 24 h. During sequencing, base calling was performed using the MinKNOW software (v19.06.8).

### Data Analysis

A pipeline was specifically developed for analyzing the data produced by MinION sequencing (Figure 1B). Raw electric signals classified as “pass” signals were converted to raw reads in the FASTQ format using MinKNOW. Average read quality was determined using FASTQC (v0.11.6). The filtering of parameters was performed based on quality and read length, and trimming was performed using Nanofilt (v2.6.0). A cutoff value of 10 was set as an acceptance quality score. The 50 nucleotide long reads were trimmed from the 5′ end and filtered for average quality (quality score >10) and size (>100 nt). We mapped the quality-controlled reads to a human *BMPR2* region (GRCh38/hg38, chr2: 202371789-202568449) using the Minimap2 program (v2.13-r850). These unique, mapped reads were extracted and converted to another format for mutation analysis using Samtools (v1.9). Single nucleotide variations (SNVs) and small insertions and deletions (Indels) were identified using Varscan (v2.4.3). Variant calls were filtered using the following parameters: a minimum coverage of 2,000 reads, an allele frequency of at least 10%, *p*-value < 0.05, and a fraction of reads from each strand (plus/minus) with a range of 0.1–10.

Then, the filtered variants were manually compared to a population database of genomic variations detected by the whole genome sequencing of Japanese individuals (https://jmorp.megabank.tohoku.ac.jp) and a database of known *BMPR2* variants (https://arup.utah.edu/database/BMPR2/BMPR2_welcome.php).

### Statistic Evaluation and Sequencing Data Visualization

Average read lengths were calculated using awk commands. The distribution of quality and length (using a Kernel density estimation) was performed using the ggplot2 (v3.2.1) package of R software.

### Sanger Sequencing

All five samples were analyzed for *BMPR2* mutation by Sanger sequencing. Primers were designed using Primer BLAST. Genomic DNA was amplified by PCR using a T100 Thermal Cycler (BIO-RAD) and TaKaRa Ex Taq DNA Polymerase (TAKARA BIO INC). Primer sequences and PCR conditions are available upon request. PCR products were subjected to nucleotide sequencing by a commercial sequencing service (FASMAC, Japan). Nucleotide sequences were compared with human reference genome sequences. Electropherograms were analyzed by visual inspection and the ATGC Software (GENETYX).

## Results

MinION sequencing runs were performed on PCR products generated by long-PCR. Five runs were carried out on five different flow cells. A summary of the number and length of reads, the quality score and the average per-base coverage for each experiment is detailed in Table 1.

**Table 1:** Read statistics derived from MinION sequencing data for each case.

**Case**	**#1**	**#2**	**#3**	**#4**	**#5**
Median length (bp)	3,524	3,454	3,244	3,120	3,465
1^st^ Quartile length (bp)	1,653	1,450	1,297	1,225	1,462
3^rd^ Quartile length (bp)	5,681	5,133	4,884	4,923	5,127
Mean quality score	20.26	19.26	18.80	18.09	18.93
Base called reads *n*	2,638,348	3,017,449	2,297,139	3,126,718	2,268,263
Reads after filtering *n* (%)	2,219,645 (84%)	2,453,722 (81%)	1,802,236 (78%)	2,283,628 (73%)	1,746,233 (77%)
Reads after alignment *n* (%)	1,550,471 (59%)	1,683,653 (56%)	975,403 (42%)	1,520,099 (56%)	1,061,661 (47%)
Average sequencing depth	135,603	133,736	80,375	119,464	106,229

*Reads were base-called using MinKNOW and filtered and trimmed using Nanofilt before alignment. n, number of reads; %, percentage of reads after quality filtering/trimming or after alignment is indicated in brackets. The average sequencing depth corresponds to the average number of reads per base*.

**Figure 2:**
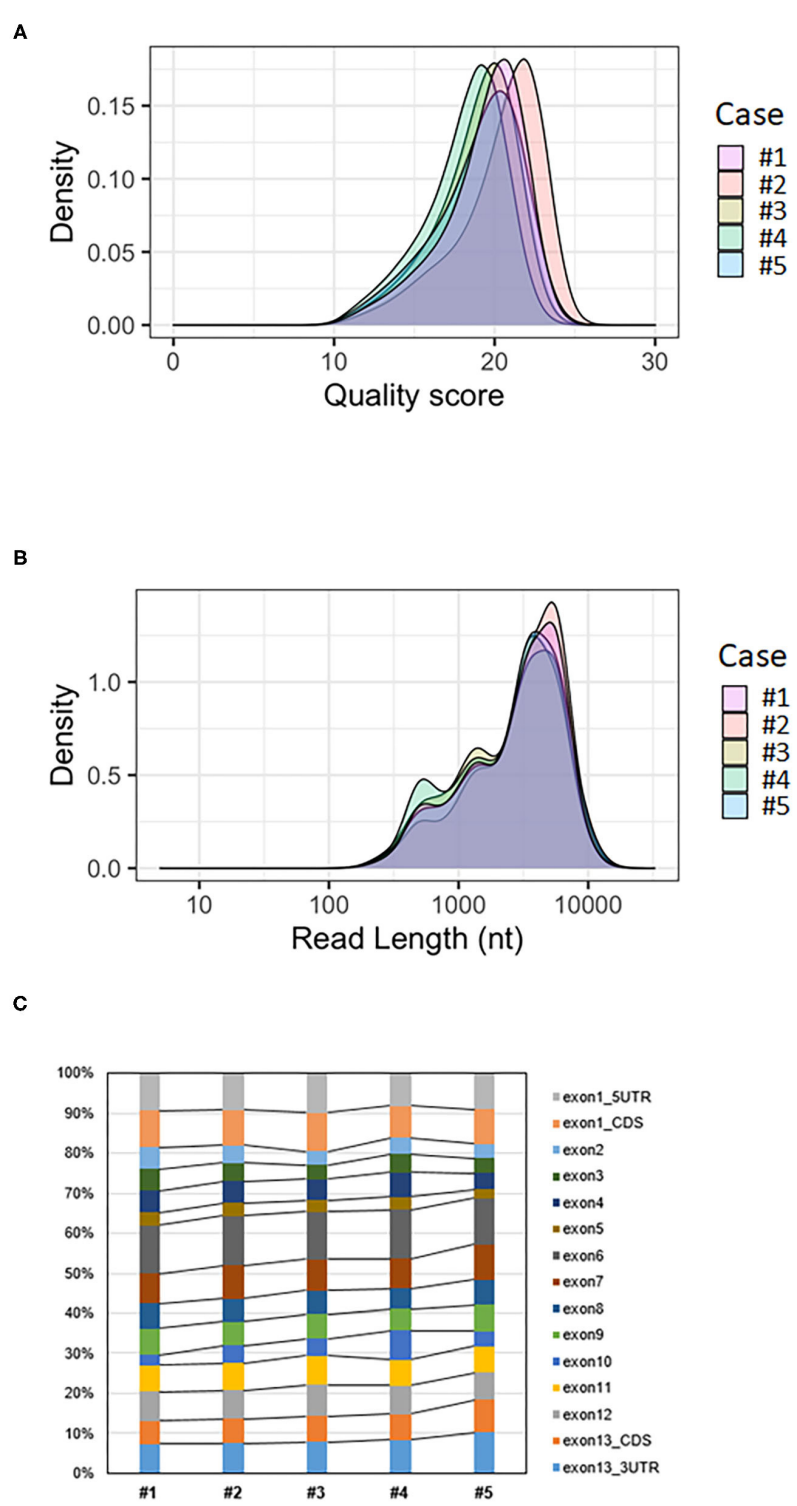
Analysis of *BMPR2* targeted MinION sequencing data. **(A)** Quality score distribution of reads. **(B)** Read length distribution of reads; the X-axis is log10 transformed; nt = nucleotides. **(C)** Ratio of aligned reads per exon.

The mean quality score of approximately 19 was considered a good score (Table 1). The plotting of the quality score distribution for the five runs showed that the quality was consistent from one flow cell to another (Figure 2A).

The median read length was approximately 3.4 kb (Table 1). Upon plotting the distribution of read lengths obtained for the five runs, we could observe that the range of read lengths was in agreement with the physical size of the sequencing library, and the amplicon size ranged from 430 bp to 9 kb (Figure 2B).

The total number of base-called reads was significantly uniform between cases and ranged from 2,268,263 to 3,126,718 reads (Table 1). After quality filtering and trimming (quality score >10, sequences shorter than 100 nucleotides removed, first 50 nucleotides of all reads trimmed), this number was reduced, and ranged from 73 to 84% of base-called reads. Finally, we mapped an average of 51% of the base-called reads (42–59%) to the reference genome after alignment (Table 1).

The average coverage was considerably uniform, with a similar number of per-base reads across flow cells, ranging from 80,375 to 135,603 reads per base (Table 1). Among aligned reads, the ratio of reads assigned to each exon (from exon 1 to exon 13) was consistent across cases (Figure 2C).

Following variant calling with VarScan's filtering parameters, we detected mostly SNVs and fewer Indels. All the variants identified for each case are listed in Table 2. These variants were interpreted based on their genomic location (exonic, splicing variant, or intronic), functional effect (synonymous or non-synonymous), and data obtained from a Japanese population database and a *BMPR2* variant database. Most variants were identified in intronic regions. No splicing variants and Indels were located within coding sequences. Sanger sequencing could not confirm the presence of Indel variants and resulted in false positive results. We checked the genomic sequences flanking these called Indels and observed nucleotide sequence repeats. These indel errors were probably related to these homopolymeric regions.

**Table 2:** Number and type of all *BMPR2* variants detected using MinION sequencing.

**Case**	**Patient background**	**SNVs**					**SNVs identified in Japanese database**
		**Total**	**CDS**	**UTR**	**Splice site**	**Intron**	
#1	PAH	32	2	3	0	27	18
#2	PAH	30	1	1	0	28	10
#3	PAH	23	2	0	0	21	9
#4	Unaffected	25	0	1	0	24	4
#5	Unaffected	28	2	0	0	26	8
**Case**	**Patient background**	**Indels**					**Indels identified in Japanese database**
		**Total**	**CDS**	**UTR**	**Splice site**	**Intron**	
#1	PAH	0	0	0	0	0	0
#2	PAH	3	0	0	0	3	0
#3	PAH	0	0	0	0	0	0
#4	Unaffected	2	0	1	0	1	0
#5	Unaffected	1	0	0	0	1	0

*Variants were manually checked for redundancy with respect to variants in an available Japanese database providing genomic variation data of whole genome sequencing of Japanese individuals (https://jmorp.megabank.tohoku.ac.jp). SNV, Single nucleotide variant; CDS, coding sequence; PAH, pulmonary arterial hypertension; UTR, untranslated region; Indel, short insertions or deletions*.

Finally, MinION sequencing revealed three mutations and two polymorphisms in four of the five cases (Cases #1, #2, #3, and #5). All these variants were validated by Sanger sequencing. Only cases #1, #2, and #3 were diagnosed with PAH. A final summary of the mutations and polymorphisms detected by MinION and Sanger sequencing has been shown in Table 3.

**Table 3:** Description and annotation of *BMPR2* mutations and polymorphisms detected using MinION sequencing for each case.

**Case**	**Location**	**Nucleotide change**	**Variant type**	**Protein change**	**Mutation type**	Clinical significance^*^	Reference SNP report^**^
#1	Exon 12	c.2379 A>C	SNV	p.Thr793=	–	Benign	rs3731697
#1	Exon 12	c.2811 G>A	SNV	p.Arg937=	–	Benign	rs1061157
#2	Exon 12	c.2695 C>T	SNV	p.Arg899XTer	Nonsense	Pathogenic	–
#3	Exon 3	c.276 A>C	SNV	p.Gln92His	Missense	Benign	–
#3	Exon 12	c.2617 C>T	SNV	p.Arg873XTer	Nonsense	Pathogenic	–
#5	Exon 3	c.276 A>C	SNV	p.Gln92His	Missense	Benign	–
#5	Exon 12	c.2617 C>T	SNV	p.Arg873XTer	Nonsense	Pathogenic	–

*All variants were validated by Sanger sequencing. SNV, Single nucleotide variant; SNP, single nucleotide polymorphism.*

* *in the database of known BMPR2 variants (https://arup.utah.edu/database/BMPR2/BMPR2_welcome.php).*

** *in the Japanese database (https://jmorp.megabank.tohoku.ac.jp)*.

Case #1 presents two variants in the coding sequence of exon 12, c.2379A>C (p.Thr793=) and c.2811G>A (p.Arg937=). These two synonymous variants are considered as benign polymorphisms and are registered in the NCBI dbSNP database (rs3731697 and rs1061157, respectively). No pathogenic mutation was identified for this case.

Case #2 was heterozygous for a nonsense mutation c.2695C>T (p.Arg899Ter) in exon 12. This variant was considered to be pathogenic in the disease database.

**Figure 3:**
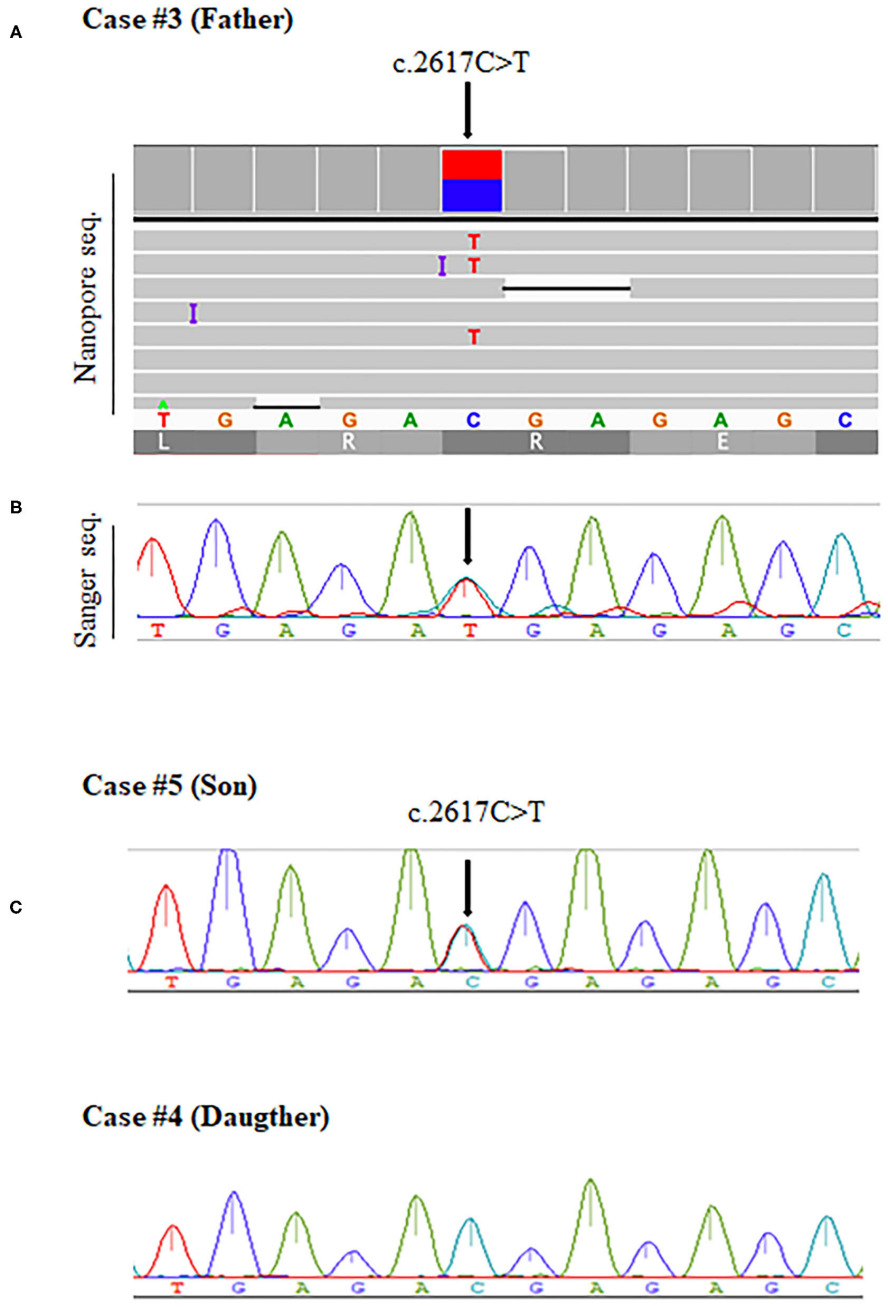
The *BMPR2* mutation c.2617C>T was detected in two members of a family (Cases #3 and #5) and validated by Sanger sequencing. Arrows indicate mutations in the heterozygous state. **(A)** Integrative genomics viewer (IGV) screenshot and Sanger validation of case #3 with PAH. **(B)** Sanger sequencing results show the same mutation being detected for case #5, the unaffected son. **(C)** Sanger sequencing results for case #4, the healthy daughter, show no mutations.

Case #3 is heterozygous for a nonsense mutation c.2617 C>T (p.Arg273Ter) in exon 12 (Figure 3A; Supplementary Figure 1). The same mutation was identified in his unaffected son (#5), who exhibited no clinical features of the disease (Figure 3B; Supplementary Figure 1). His healthy daughter (#4) did not carry this mutation (Figure 3C; Supplementary Figure 1). This variant is considered pathogenic in the disease database. The father (#3) and his son (#5) also presented with a missense variant c.276 A>C (p.Gln92His) located in exon 3. The significance of this variant is considered to be uncertain.

## Discussion

Here, we developed a protocol and performed a pipeline analysis via the targeted nanopore sequencing of *BMPR2*. Nanopore sequencing has been successfully used to sequence genomes of many species (viruses, bacteria, animals, plants, and humans), but limited data are available regarding the use of this technology in clinical settings (15–22). To the best of our knowledge, the MinION platform has not been applied for PAH diagnosis.

Sequencing is essential for determining the genetic basis of pathogenicity and for genetic counseling. Current Sanger technology is time consuming and labor intensive. New sequencing technologies have revolutionized DNA sequencing. However, though NGS generally requires a massive investment in capital equipment, its use could be limited in small laboratories, owing to its high cost and high input material requirements. Another limitation of second-generation platforms is their short-read lengths, which range from 150 to 300 bp (12, 23). The nanopore sequencing platform is a third-generation platform that generates long read lengths; its sequencing process occurs in real-time (13, 14). The MinION nanopore sequencer is a small, easy to manipulate, and affordable device, capable of sequencing many gigabases (Gb) in a single experiment (24). This portable device allows sequencing to be performed in a small laboratory or research unit, where the large-scale installation of sequencing equipment is unfeasible.

We are the first to report about the use of MinION sequencing for the rapid genetic analysis of PAH patients. *BMPR2* was focused upon because its frequency was the highest. Heterozygous germline mutations in *BMPR2* represent the central susceptibility factor in the precipitation and progression of PAH (9). Our laboratory has developed a pipeline for rapidly sequencing human samples and validating them via their comparison with standard Sanger sequencing results.

To evaluate the potential of MinION sequencing for genetic analysis, we analyzed a small set of five samples. We have discussed the ability of MinION to detect *BMPR2* variants below.

We identified two polymorphisms and three mutations, comprising two nonsense mutations considered to be pathogenic, and one missense mutation (see below). Both MinION sequencing and Sanger sequencing have detected these *BMPR2* variants, which were located in exonic regions. There was no discrepancy.

Two polymorphic variants in exon 12 (c.2379 A>C and c.2811 G>A), observed in Case #1, caused non-synonymous changes in codons and did not alter amino acid sequences (p.Thr793= and p.Arg937=). These polymorphic variants were reportedly observed in unaffected individuals (25, 26) and were not expected to be clinically significant.

The nonsense mutation c.2695C>T (p.Arg899Ter) in exon 12 (Case #2) has been previously reported in individuals with PAH (6, 25–27). In the family mentioned above, we could identify a nonsense mutation c.2617 C>T (p.Arg273Ter) in the affected father (Case #3) and his son (Case #5), who did not exhibit symptoms yet. This mutation was previously reported to be pathogenic and showed reduced penetration among carriers (5, 26). The disease onset varied widely between individuals who harbored the same gene defect within a family, suggesting that other factors, genetic and/or environmental, were important for disease progression (10, 26). Furthermore, it is essential to identify this mutation for clinical management; the necessity to do so extends beyond the needs of the affected patient, as it enables earlier disease diagnosis and adoption of preventive measures in carriers (28).

In the same family, we also identified a c.276 A>C (p.Gln92His) missense variant in the father and son. This mutation was classified as a benign mutation in our *BMPR2* database (http://arup.utah.edu/database/BMPR2/BMPR2_welcome.php, last update January 2013). However, this missense variant is listed in the ClinVar database (http://www.ncbi.nlm.nih.gov/clinvar, last evaluated March 2016) and interpreted as a variant with uncertain significance, as reported previously (29, 30). It should be noted that several years have elapsed since variant interpretation was performed in our *BMPR2* database. Changes in variant classification might have occurred over time due to the advent of NGS and the detection and interpretation of numerous variants (31). Hence, special care is warranted while determining their possible pathogenicity. Nevertheless, in this case, the change in variant classification did not lead to a clinically significant change.

Here, we demonstrated the feasibility of using MinION sequencing as a tool for genetic analysis in patients with PAH. However, there are some limitations, including Indel variant calling-related errors. It is well-known that the process of nanopore sequencing of homopolymeric regions is difficult and error prone (19, 20, 32). Improvements are essential to enable accurate indel calling. The sample size of our study is small; however, given the satisfactory results obtained, further studies should be performed to analyze more cases.

One advantage is that there is no instrument cost with a cheap starter kit (including MinION device, two flow cells and library preparation kit) costing approximately US$ 1,000. Then single extra flow cell costs US$ 500–900 (depending on the number purchased) and reagents cost approximately US$ 100 per sample. To increase cost-effectiveness, sample multiplexing would be necessary. Moreover, ongoing developments in the flow cell design have contributed to cost-efficient sequencing with the introduction of Flongle (Flow cell Dongle). Flongle is an adapter for MinION that enables DNA sequencing on smaller, lower cost (US$ 90 per flow cell), single-use flow cells. Thus, the lower cost per sample makes Flongle more suitable for targeted sequencing and smaller tests.

Finally, we have focused on *BMPR2* alone, but the analysis of a panel of genes implicated in PAH is a challenge, and should be attempted in future studies.

## Conclusion

MinION sequencing can be used to detect *BMPR2* mutations in patients with PAH. The MinION device is easy to use and provides long sequence reads. Thus, it can be concluded that the MinION nanopore sequencer is a promising tool for screening *BMPR2* mutations.

## Data Availability Statement

The sequencing data presented in this study are deposited in DNA Data Bank of Japan, accession number: DRA012598.

## Ethics Statement

The studies involving human participants were reviewed and approved by institutional ethics committee at the International University of Health and Welfare (approval number 5-16-25). The patients/participants provided their written informed consent to participate in this study. Written informed consent was obtained from the relevant individual(s), and/or minor(s)' legal guardian/next of kin, for the publication of any potentially identifiable images or data included in this article.

## Author Contributions

YT takes responsibility for the content of the manuscript, including the data, and analysis. YT and TT conceived and designed the study. TT performed nanopore sequencing, bioinformatics analysis, and primary data analysis. SB and YT participated in data analysis and interpretation. AF, HT, RT, and AK contributed to sample collection. SB prepared the first draft of the manuscript, along with TT. YT supervised the manuscript preparation process. All authors reviewed the content of the draft, approved the final manuscript, and agreed to be accountable for all aspects of the work.

## Funding

This work was supported by MEXT KAKENHI, via grant number JP18K08183, and the Actelion Academia Prize and GSK Japan Research Grant 2018.

## Conflict of Interest

This work was supported by MEXT KAKENHI, via grant number JP18K08183, and the Actelion Academia Prize and GSK Japan Research Grant 2018. Both Actelion Academia Prize and GSK Japan Research Grant are independent from industrial activities and totally funded on scientific activities. These funds contributed only our experimental procedures and the preparation of manuscripts. The authors declare that the research was conducted in the absence of any commercial or financial relationships that could be construed as a potential conflict of interest.

## Publisher's Note

All claims expressed in this article are solely those of the authors and do not necessarily represent those of their affiliated organizations, or those of the publisher, the editors and the reviewers. Any product that may be evaluated in this article, or claim that may be made by its manufacturer, is not guaranteed or endorsed by the publisher.

## Abbreviations

*BMPR2*: bone morphogenetic protein receptor type 2
NGS: Next generation sequencing
PAH: pulmonary arterial hypertension
PCR: polymerase chain reaction
SNV: Single nucleotide variant.
