# Supplementary material for: Case Report: BMPR2-Targeted MinION Sequencing as a Tool for Genetic Analysis in Patients With Pulmonary Arterial Hypertension
Source: Front Cardiovasc Med. 2021 Sep 13;8:711694. doi: 10.3389/fcvm.2021.711694 (PMC8473694; doi:10.3389/fcvm.2021.711694)
Supplement: Supplementary file 1 [file Table_1.DOCX]

**Supplementary Table 1.** Primer sequences and PCR conditions for long-PCR amplification of *BMPR2*

| **No** | **Exon** | **Product size (bp)** | **Annealing temperature (°C)** | **Primer sequence** | |
| --- | --- | --- | --- | --- | --- |
| 1 | Exon 1 | 6018 | 64.1 | F | TTTCTGTTGGTGCTGATATTGCCATACTAAAATCCAATTTCTCTGAGTGCTA |
|  |  |  |  | R | ACTTGCCTGTCGCTCTATCTTCATTATTTAAAACGCATTACAGAAGTTGACC |
| 2 | Exon 1 | 6501 | 67.3 | F | TTTCTGTTGGTGCTGATATTGCAATTATCAGTATACTTGAGGGACTTTTTCC |
|  |  |  |  | R | ACTTGCCTGTCGCTCTATCTTCGAAATGAAAATATCCAAAGGAAAGGATGAC |
| 3 | Exon 2,3 | 6331 | 61.8 | F | TTTCTGTTGGTGCTGATATTGCTTGTCTTTACTTAACCCAATCTCTTCATTT |
|  |  |  |  | R | ACTTGCCTGTCGCTCTATCTTCCCATTGTTAGGTACTTAATTTTGTTTGCTT |
| 4 | Exon 4, 5 | 6376 | 67.3 | F | TTTCTGTTGGTGCTGATATTGCTAAATCTTACAGCTAACTTGGGAAATACAG |
|  |  |  |  | R | ACTTGCCTGTCGCTCTATCTTCCTCAAAGTGCTTATCTACAAACTATTACCA |
| 5 | Exon 6, 7 | 5233 | 64.1 | F | TTTCTGTTGGTGCTGATATTGCCTTTGATTAGAGATTTGTACAGGGTATGAG |
|  |  |  |  | R | ACTTGCCTGTCGCTCTATCTTCACCTAATAGAAAGACAGTGACTAAGATTCA |
| 6 | Exon 8,9 | 5117 | 67.3 | F | TTTCTGTTGGTGCTGATATTGCTGTTATTACTTATTCAGCTGTTTGTTGCTT |
|  |  |  |  | R | ACTTGCCTGTCGCTCTATCTTCCATAAATTTGTCCAATGCTAGTTCCTTTTT |
| 7 | Exon 10 | 430 | 61.8 | F | TTTCTGTTGGTGCTGATATTGCTCTGTCATTCTTTTCTACAAATCCAC |
|  |  |  |  | R | ACTTGCCTGTCGCTCTATCTTCCAGCTGTTACCTAATCTGGCAA |
| 8 | Exon 11, 12 | 6161 | 64.1 | F | TTTCTGTTGGTGCTGATATTGCACTGTTTTTGATACTACTGTTTTGGAACTA |
|  |  |  |  | R | ACTTGCCTGTCGCTCTATCTTCTTCTGACTATTGTGACACTGACTTTTTAAT |
| 9 | Exon 13 | 9067 | 61.8 | F | TTTCTGTTGGTGCTGATATTGCAGATCGCTACCAAAACCAGAAGAT |
|  |  |  |  | R | ACTTGCCTGTCGCTCTATCTTCTCTTGCCACAAGGAGAGCTTG |
