# Supplementary figures and images for: Case Report: BMPR2-Targeted MinION Sequencing as a Tool for Genetic Analysis in Patients With Pulmonary Arterial Hypertension
Source: Front Cardiovasc Med. 2021 Sep 13;8:711694. doi: 10.3389/fcvm.2021.711694 (PMC8473694; doi:10.3389/fcvm.2021.711694)

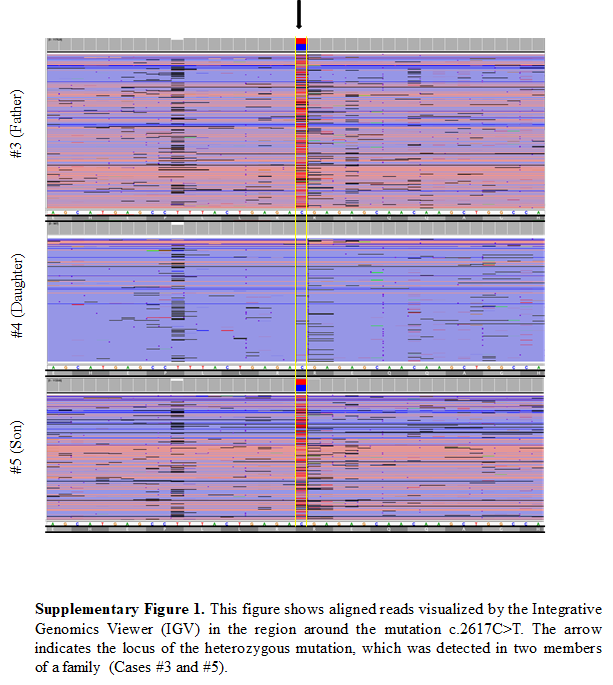

Supplement: Supplementary file 2 [file Image_1.TIF]
